# Prevention of High-Dose Cytosine Arabinoside-Induced Acute Pericarditis with Preemptive Dexamethasone Administration: A Case Report and Literature Review

**DOI:** 10.1155/2018/4726451

**Published:** 2018-02-11

**Authors:** Weerapat Owattanapanich, Theera Ruchutrakool

**Affiliations:** Division of Hematology, Department of Medicine, Faculty of Medicine, Siriraj Hospital, Mahidol University, Bangkok, Thailand

## Abstract

Pericarditis/pericardial effusion (PC/PEEF) is a rare but fatal complication of cytosine arabinoside (Ara-C). We report an acute myeloid leukemia (AML) patient who developed massive pericardial effusion after a second Ara-C exposure. As Ara-C was most beneficial in controlling the leukemia, she was treated with a further cycle of Ara-C along with dexamethasone to prevent the complication from reoccurring. No PC/PEEF was subsequently detected.

## 1. Introduction

AML is the most common acute leukemia in adults. Its annual incidence in Thailand is 1.4 per 100,000 population, with 5-year disease-free survival and overall-survival rates of 41.0% and 22.2%, respectively [1]. The most efficient chemotherapeutic agents are anthracyclin and Ara-C. Ara-C is administered as a standard dose during induction and a high dose during consolidation. Besides bone marrow suppression, nausea, vomiting, fatigue, and mouth sores, the side effects of high-dose Ara-C (HIDAC) include conjunctivitis and cerebellar toxicity. PC/PEEF, a rare but often lethal complication of standard and high-dose Ara-C, was first reported in 1984 [2]. No management guidelines exist for PC/PEEF. Based on a literature review and our experience with an AML patient with this rare complication after Ara-C treatment, we propose a strategy to manage it and rechallenge with Ara-C without the complication.

## 2. Case Report

A 38-year-old female presented with fatigue for 2 months. On examination, pale skin and petechiae along both legs were observed; others were unremarkable. The initial blood count showed a hemoglobin of 7.8 g/dL, WBC of 242.7 × 10^9^/L (myeloblasts and monoblasts constituted over 90%), and platelet count of 21 × 10^9^/L. By flow cytometry, the malignant cells (86%) were positive for CD13, CD33, CD11b, CD11c, CD14, CD64, CD117, and MPO which were compatible with AML-M4 according to FAB classification. By G-banding karyotyping, no abnormalities were observed. After establishing the diagnosis of AML, the patient received standard remission induction therapy with Ara-C (100 mg/M^2^/day; 12-hour continuous infusion once daily for 7 consecutive days) and idarubicin (12 mg/M^2^/day; intravenous infusion for 3 consecutive days). She regenerated at day 24 and entered complete remission without grade 4 complications except cytopenia. It was planned that she would receive allogeneic stem cell transplantation. While awaiting approval of this treatment from her covered medical expenses, we decided to control her disease with consolidation therapy, HIDAC regimen for 3 cycles.

**Figure 1:**
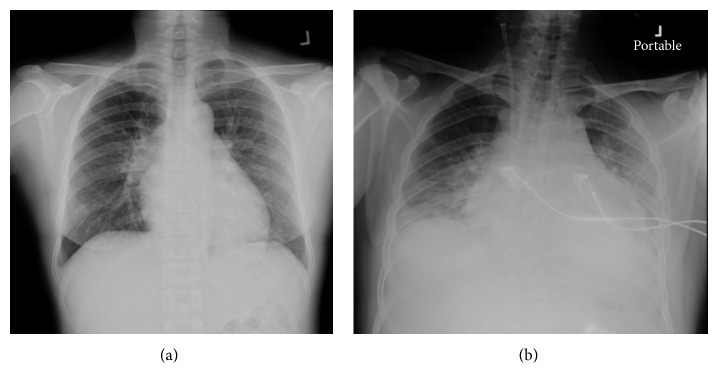
Chest X-rays: (a) prior to cytosine arabinoside; (b) day 3 of the first cycle of HIDAC.

A week after complete remission, Ara-C (1,000 mg/M^2^/day; 2-hour continuous infusion every 12 hours for 6 doses) commenced. On day 3 of this regimen, during administration of the fourth Ara-C dose, she complained of a sudden onset of dyspnea and sharp, persistent pain in the substernal and epigastric areas. She had no fever, sweating, cough, or palpitation. Her temperature was 36.8°C and pulse rate was 80/min and regular, but her blood pressure, measured repeatedly at all extremities, was 80/40 mmHg. A chest X-ray revealed massive cardiomegaly (Figure 1(b)) relative to the baseline cardiac size on the admission day (Figure 1(a)). Electrocardiography showed normal sinus rhythm and no low voltage, electrical alternans, or ST-T abnormality.

An echocardiography disclosed massive PEEF without cardiac tamponade. The left ventricular ejection fraction was 71% without either an abnormal wall motion or significant valve dysfunction. Computed tomography of the chest and abdomen was unremarkable except revealing massive PEEF. Troponin-T was under 0.003 ng/mL (<0.01 ng/mL), and CK-MB was 0.67 IU/L (5–25 IU/L). Viral serologic studies (herpes simplex virus, cytomegalovirus, Epstein–Barr virus, and hepatitis B virus) detected by IgM and IgG were all negative. Antinuclear antibody was also negative.

Given no other apparent causes of PC/PEEF, Ara-C-induced PC/PEEF was highly suspected. After 2,000 mL of normal saline infusion within 15 minutes, hypotension was unimproved. The initial central venous pressure revealed 24 cm H_2_O. Dopamine (10 *µ*g/kg/minute) was started. Besides ceasing Ara-C, 10 mg/day of intravenous dexamethasone was initiated. The substernal and epigastric pain subsequently disappeared within six hours, with BP stabilized at 110/80 mmHg. The inotropic drug was discontinued within 26 hours. The cardiac shadow was markedly decreased three days after commencement of the dexamethasone treatment. At day 7, an echocardiography revealed only small pericardial effusion, so a planned pericardiocentesis was canceled. The dexamethasone was tapered and stopped by day 11. She was discharged on day 23, still without evidence of effusion.

As Ara-C was deemed essential for the AML treatment, it was decided to repeat the HIDAC consolidation therapy but with preemptive, high-dose dexamethasone. Ten mg/day intravenous dexamethasone was therefore initiated the day before cytoreduction and continued for 6 days of each cycle. The HIDAC regimen administration went without complications. The patient successfully underwent a matched-sibling stem cell transplantation in July 2012. As of January 2017, she remains in remission.

## 3. Discussion

PC/PEEF is an uncommon, but potentially fatal, AML complication. Its pathogenesis is multifactorial, possibly resulting from the underlying disease, the concomitant infection, accompanying bleeding complications arising from thrombocytopenia or DIC, the chemotherapy, or radiation. Thus, Ara-C-induced PC/PEEF should always be an exclusion diagnosis. In our case, we excluded both virological and autoimmune pathogenesis. Despite the lack of cytology of the pericardial effusion, we deemed that leukemic involvement and hemorrhagic complications in this patient, who was in remission and without any complaints, could be ruled out. Thus, this severe episode should be described to the cytoreductive regimen. Chemotherapeutic agents which have been reported to induce PC/PEEF include daunorubicin, doxorubicin, cyclophosphamide, bleomycin, and actinomycin D. Our patient received anthracycline six weeks before the development of PC/PEEF; the longest duration of anthracycline-induced PC/PEEF onset ever reported was 29 days. Therefore, Ara-C is the most likely cause of PC/PEEF in our patient, rather than doxorubicin.

Ara-C-induced PC/PEEF was first described by Vaickus in 1984 [2]; a literature review revealed that only 10 patients (including our patient) have been reported. Interestingly, two patients developed two episodes of this complication [3, 4]. The pathogenesis of these severe complications is unclear, but type IV delayed hypersensitivity [4–9], anaphylaxis [10], and direct toxicity to the endothelium [11] have been suggested.

Table 1 tabulates previously published series of PC/PEEF. The onset of PC/PEEF varied between 2 and 29 days (median: 3 days) after receiving repeated doses of Ara-C. Most patients received the HIDAC regimen. Our patient developed PC/PEEF three days after HIDAC; we hypothesize that there was a type IV delayed hypersensitivity, compatible with such reactions developing within 10 days of the last exposure. To prove this, a cardiac muscle biopsy showing infiltration of T-lymphocytes would be required.

These considerations leave us with the question of how to treat PC/PEEF, either prophylactically or upon occurrence. PEEF management depends on the degree of cardiac tamponade; if clinical cardiac tamponade is detected, pericardiocentesis is the treatment of choice. Systemic corticosteroids were first introduced in treating this complication in 1984 [2] with the hypothesis of a delayed hypersensitivity or anaphylaxis, but the response was undesirable. This could be due to an insufficient amount of steroid being used (prednisolone 20 mg/day). Subsequently, Gillis introduced higher dose of steroid (hydrocortisone 200 mg/day), and the results were promising [5], similar to our patient. Successful results with various types and dosage of corticosteroids, such as intravenous hydrocortisone (200 mg/day) and oral methylprednisolone (0.5 mg/kg/day), have been reported [6–8]. Furthermore, we insisted on using the HIDAC regimen for the second course of consolidation chemotherapy with prophylactic dexamethasone. As PC/PEEF did not reappear, we suggest that patients with Ara-C-induced PC/PEEF may be treated with the same regimen combined with a systemic high-dose corticosteroid. From our experience, the optimal dexamethasone dosage to prevent this complication is at least 8–10 mg/day, which is equivalent to hydrocortisone dose of 200–250 mg/day or prednisolone dose of 40–50 mg/day.

## Figures and Tables

**Table 1:** Reported cases of Ara-C-induced pericarditis/pericardial effusion.

Author	Year	Patients/disease	Site of complication	Chemotherapy regimen and onset of event (day after receiving chemotherapy)	Treatment of pericarditis or pericardial effusion	Results
Vaickus and Letendre	1984	25-year-old man with relapsed ALL	PC/PEEF with cardiac tamponade	Day 2 after the fourth dose of the second cycle of high-dose Ara-C (3 g/M^2^ for 10 doses/cycle) (consolidation)	Prednisolone 10 mg twice daily and salicylate 500 mg twice daily for 2 days, but no response; pericardiocentesis was performed	Full recovery after pericardiocentesis
Gillis et al.	1992	51-year-old woman	PC with minimal PEEF	Day 3 after the fifth dose of the first cycle of high-dose Ara-C (3 g/M^2^ for 12 doses/cycle) (consolidation)	Hydrocortisone 200 mg once daily intravenously	Full recovery within a week without pericardiocentesis
Reykdal et al.	1995	47-year-old man with AML-M4	PC, no PEEF	Day 11 after the last dose of the first cycle of high-dose Ara-C (2 g/M^2^ every 12 hours for 6 days) (consolidation)	Supportive treatment with NSAIDs	Pain relieved by NSAIDs within a day
Hermans et al.	1997	37-year-old man with AML-M2	PC/PEEF	Day 4 after the 5th dose of 3rd cycle of high-dose Ara-C (3 g/M^2^ every 12 hours for 4 days) (consolidation)	Methylprednisolone 0.5 mg/kg orally for 8 days, and tapered off at day 11	Complete recovery by day 7 of methylprednisolone
Yamada et al.	1998	61-year-old man with AML-M2 and 65-year-old man with MDS-related AML-M2	PEEF	Relapsed AML, low-dose Ara-C (induction remission) AML-M2 low-dose Ara-C (consolidation)	Pulse methylprednisolone plus pericardiocentesis	Not reported
Woods et al.	1999	47-year-old man with AML-M2	Large PEEF	Day 9 after the first induction remission with daunorubicin and Ara-C (100 mg/M^2^ D1-7); then, high-dose Ara-C (2 g/M^2^ on D8-10) (induction remission)	Pericardiocentesis	Recovery by 6 weeks
Gähler et al.	2003	64-year-old man with AML-M4	1st: PC/small PEEF	1st episode: day 29 after 7 + 3 regimen (200 mg/M^2^ Ara-C) (induction remission)	1st: NSAIDs	Recovery in 48 hours
			2nd: PC with PEEF and cardiac tamponade	2nd episode: day 3 after high-dose Ara-C (1 g/M^2^ every 12 hours for 6 days) (consolidation)	2nd: Pericardiocentesis	Immediately improved after pericardiocentesis
Lee et al.	2011	25-year-old woman with AML-M4	Recurrent PC	1st episode: day 6 after the sixth dose of high-dose Ara-C (second cycle of induction remission)	1st: high-dose ibuprofen (1,200 mg/day)	Responded well
				2nd episode: day 2 after the sixth dose of high-dose Ara-C (first consolidation)	2nd: high-dose ibuprofen (1,200 mg/day) + colchicine (0.6 mg/day)	Responded well within 10 days
This study	2017	38-year-old woman with AML-M4	PC/PEEF	Day 3 after the fourth dose of high-dose Ara-C (first consolidation)	Dexamethasone 10 mg intravenously once daily	Complete recovery within 3 days
